# Minimal Coarse-Grained Model for Immunoglobulin G: Diffusion and Binding under Crowding

**DOI:** 10.1021/acs.jpcb.3c02383

**Published:** 2023-08-17

**Authors:** Edyta Słyk, Tomasz Skóra, Svyatoslav Kondrat

**Affiliations:** † Institute of Physical Chemistry, Polish Academy of Sciences, Warsaw 01-224, Poland; ‡ Department of Theoretical Chemistry, Institute of Chemical Sciences, Faculty of Chemistry, Maria Curie-Skłodowska University in Lublin, Lublin 20-031, Poland; § Institute for Computational Physics, University of Stuttgart, Stuttgart 70569, Germany

## Abstract

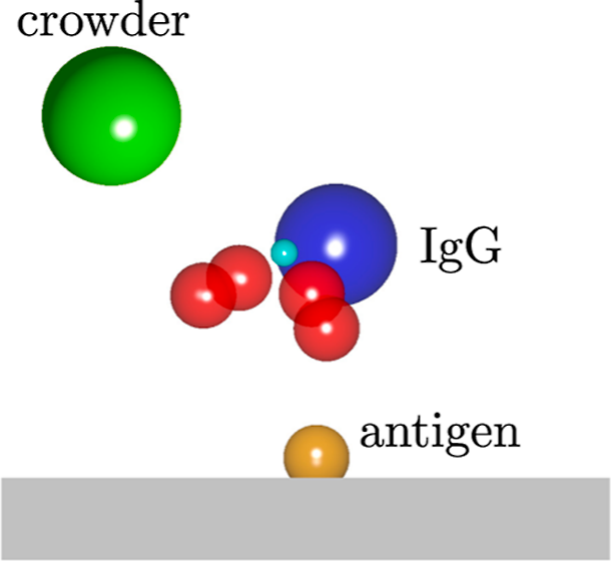

Immunoglobulin G (IgG) is the most common type of antibody found in blood and extracellular fluids and plays an essential role in our immune response. However, studies of the dynamics and reaction kinetics of IgG–antigen binding under physiological crowding conditions are scarce. Herein, we develop a coarse-grained model of IgG consisting of only six beads that we find minimal for a coarse representation of IgG’s shape and a decent reproduction of its flexibility and diffusion properties measured experimentally. Using this model in Brownian dynamics simulations, we find that macromolecular crowding affects only slightly the IgG’s flexibility, as described by the distribution of angles between the IgG’s arms and stem. Our simulations indicate that, contrary to expectations, crowders slow down the translational diffusion of an IgG less strongly than they do for a smaller Ficoll 70, which we relate to the IgG’s conformational size changes induced by crowding. We also find that crowders affect the binding kinetics by decreasing the rate of the first binding step and enhancing the second binding step.

## Introduction

Immunoglobulins are large, flexible glycoproteins and one of the most important molecules in living organisms, playing a critical role in the immune response system^1^ and finding biotechnological and biomedical applications.^2,3^ Immunoglobulins are produced by plasma cells and aid the immune system in destroying pathogens by recognizing and binding to their antigens. While antigen-binding sites come in a wide variety to recognize millions of different antigens, the remaining parts of immunoglobulins are relatively constant and occur only in a few variants. Among them, immunoglobulin G (IgG) is the most common type found in blood, representing about 75% of serum antibodies in humans.^4^

Proteins amount to about 9% of the blood plasma mass.^5^ Assuming that the average protein density is about 1.3 times larger than that of water,^6^ it translates into about 7% of the occupied volume fraction. While this is much less than typical intracellular crowding,^7,8^ the all-atom rigid-model simulations^9−11^ of IgG in physiologically relevant crowded environments are nevertheless computationally challenging owing to the immense size of IgG (about 150 kDa) and other macromolecules. Moreover, the high flexibility of IgG, reported by atomic force microscopy^12^ and cryo-electron tomography,^13^ would likely make such simulations unphysical.

Several coarse-grained models of antibodies have been developed recently. Chaudhri et al.^14^ introduced 12- and 26-bead models of two therapeutic antibodies and studied the role of interactions in their self-association.^15,16^ These authors considered only two configurations of rigid antibodies instead of accounting for their full flexibility. Galanti et al.^17^ developed a 96-bead model, finely approximating the IgG shape and adjusting the parameters to reproduce the distribution of angles measured experimentally with cryo-electron tomography.^13^ De Michele et al.^18^ took a similar route but considered a coarser model consisting of only seven beads. However, these authors used ellipsoidal beads to better approximate the IgG arms and stem, which is difficult to implement and simulate using the available simulation packages.

In this study, we propose a six-bead coarse-grained model of IgG that reproduces the distribution of angles and diffusion coefficient of IgG measured experimentally. We call it a “minimal model” as we find that reproducing the IgG flexibility and shape with fewer beads is problematic. We use this model in Brownian dynamics (BD)^19−21^ simulations to investigate how physiologically relevant macromolecular crowding affects IgG’s flexibility, diffusion, and binding kinetics. We find that the dependence of IgG diffusion on crowding differs qualitatively from the behavior expected for rigid bodies. Our simulations indicate, however, that crowders affect the angle distribution and the binding kinetics relatively weakly.

## Model of IgG

**Figure 1:**
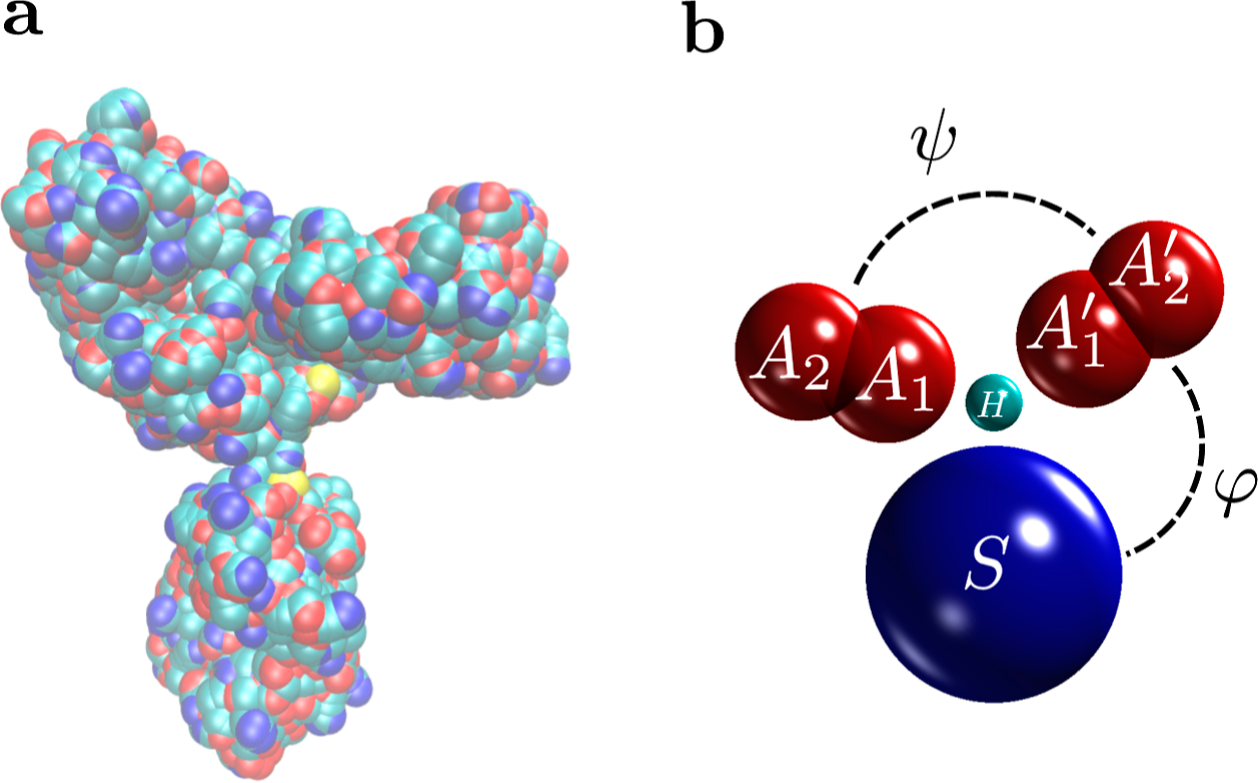
IgG. (a) Crystal structure of human IgG^22^ taken from Protein Data Bank (http://www.rcsb.org/, PDB ID: 1HZH)^23^ and visualized with VMD.^24^ (b) IgG model used in this work (see Tables 1 and 2).

IgG is a Y-shaped protein consisting of a nearly spherical stem and two elongated arms (Figure 1a); each tip of the arm has a paratope that binds to antigens on the surface of an infected cell or microbe. We model the IgG stem with a single spherical bead and approximate each IgG’s arms with two overlapping beads, connected to the stem by a small hinge enabling IgG’s exceptional flexibility, resembling a ball-and-socket joint^25^ (Figure 1b). We assume uncharged beads and choose the bead sizes to match the sizes of the corresponding IgG subunits as closely as possible and reproduce the diffusion coefficient of an IgG in infinite dilution (see below). To model the flexibility of IgG’s arms, we use bonded potentials between two neighboring beads, *i* and *j*
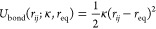
1where *r*_*ij*_ is the distance between the beads, *r*_eq_ is the equilibrium bond length, and κ is the force constant. For the angular potentials between three neighboring beads *i*, *j*, and *k*, we take
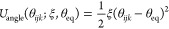
2where θ_*ijk*_ is the angle between the beads, θ_eq_ is the equilibrium angle, and ξ is the angle force constant.

We assume steric interactions between any bead of an IgG and other particles but allow for overlapping of beads within the same IgG. In this article, we neglect dispersion interactions to focus on the effects of IgG’s shape and flexibility on the dynamics and binding kinetics under macromolecular crowding.

To relate the model parameters to measurable quantities such as the IgG hydrodynamic radius and angle distributions, we performed BD simulations of a single IgG (Section S1). We accounted for hydrodynamic interactions between the beads within the Rotne–Prager–Yamakawa (far-field) approximation,^26−28^ which is essential to obtain the correct value of the diffusion coefficient.^29,30^ From the BD trajectories, we computed a mean square displacement (MSD) and the distributions of angles between the arms (ψ) and between the arm and the stem (φ). The diffusion coefficient *D*_0_ was extracted from the MSD in the usual way as *D*_0_ = MSD/6*t* in the limit of large *t*. In Tables 1 and 2, we present the values of the bead radii and interaction parameters providing the best fit to the experimental data. These values give the translational and rotational diffusion coefficients *D*_0_ = 36.80(103) nm^2^ μs^–1^ and *D*_r,0_ = 1.110(34) rad^2^ μs^–1^. From *D*_0_ and using the Stokes–Einstein–Sutherland relation, we get for the hydrodynamic radius *a*_H_ ≈ 5.8 nm, closely matching the measured value ≈6 nm.^31^

**Table 1:** Subunit Sizes of the IgG Model

subunit	*S* (stem)	*H* (hinge)	*A*_1_, *A*_2_, *A*_1_′, and *A*_2_′ (arms)
*a*_H_ (nm)	4.50	1.00	2.42

**Table 2:** Equilibrium Bond Lengths and Angles of the IgG Model

bond	bond length, *r*_eq_ (nm)	force constant, κ (kcal mol^–^^1^ nm^–^^2^)
*SH*	6.0	1909.9
*HA*_1_*HA*_1_′	3.9	1909.9
*A*_1_*A*_2_, *A*_1_′*A*_2_′	3.0	1909.9

angles	angle value, θ_eq_ (°)	force constant, ξ (kcal mol^–^^1^ rad^–^^2^)
*SHA*_1_, *SHA*_1_′	125	0.05
*HA*_1_*A*_2_, *HA*_1_*A*_2_′	180	10.00
*A*_1_*HA*_1_′	110	0.05

Parameter , introduced by Skóra et al.,^32^ quantifies how fast a particle rotates compared to its translational motion. For our IgG model, we find *k* ≈ 8.6° nm^–1^, which means that an IgG rotates on average by ≈50° when diffusing a distance equal to its hydrodynamic radius. This is relatively fast compared, e.g., with a 16 nm long double-stranded DNA, which has *k* ≈ 6.8° nm^–1^.^32^

**Figure 2:**
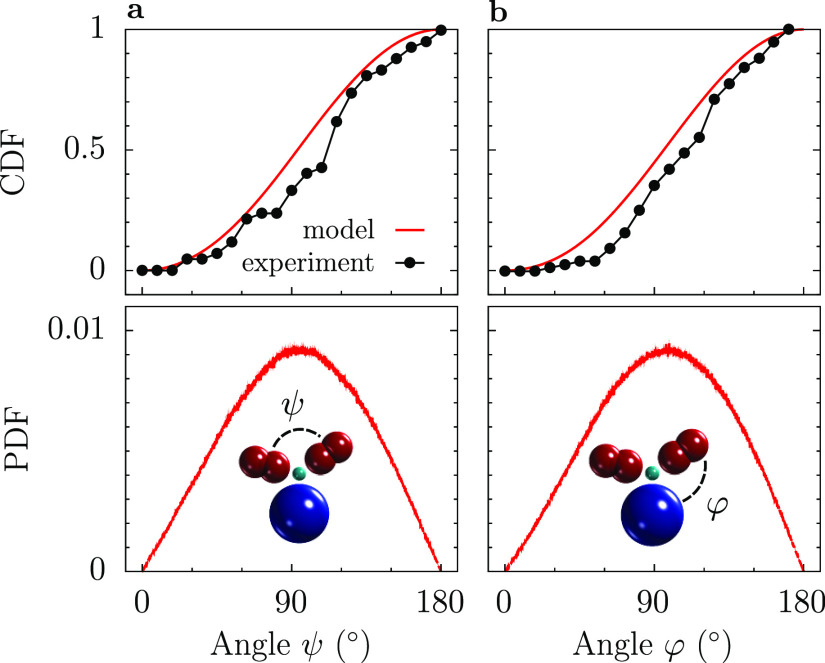
Distribution of angles in IgG from models and experiments. (a) CDF (top) and PDF (bottom) of the angle ψ between two IgG arms. (b) CDF (top) and PDF (bottom) of the angle φ between the IgG stem and arms. The symbols show the experimental data from ref (13), and the lines show the simulation results for the model IgG introduced in this work. The bottom plots do not contain experimental data because they are not provided in ref (13), and the numerical differentiation of the experimental CDFs does not lead to satisfactory results.

Figure 2 presents cumulative distribution functions (CDFs) of angles ψ and φ (top plots), showing that our model decently reproduces the experimental data by Bongini et al.^13^ The bottom plots in Figure 2 show the probability density functions (PDFs) obtained from the model CDFs by numerical differentiation. Note that the maximum of both PDFs is close to 90°, and both PDFs are only slightly skewed.

## Results and Discussion

### Arms’ Flexibility

**Figure 3:**
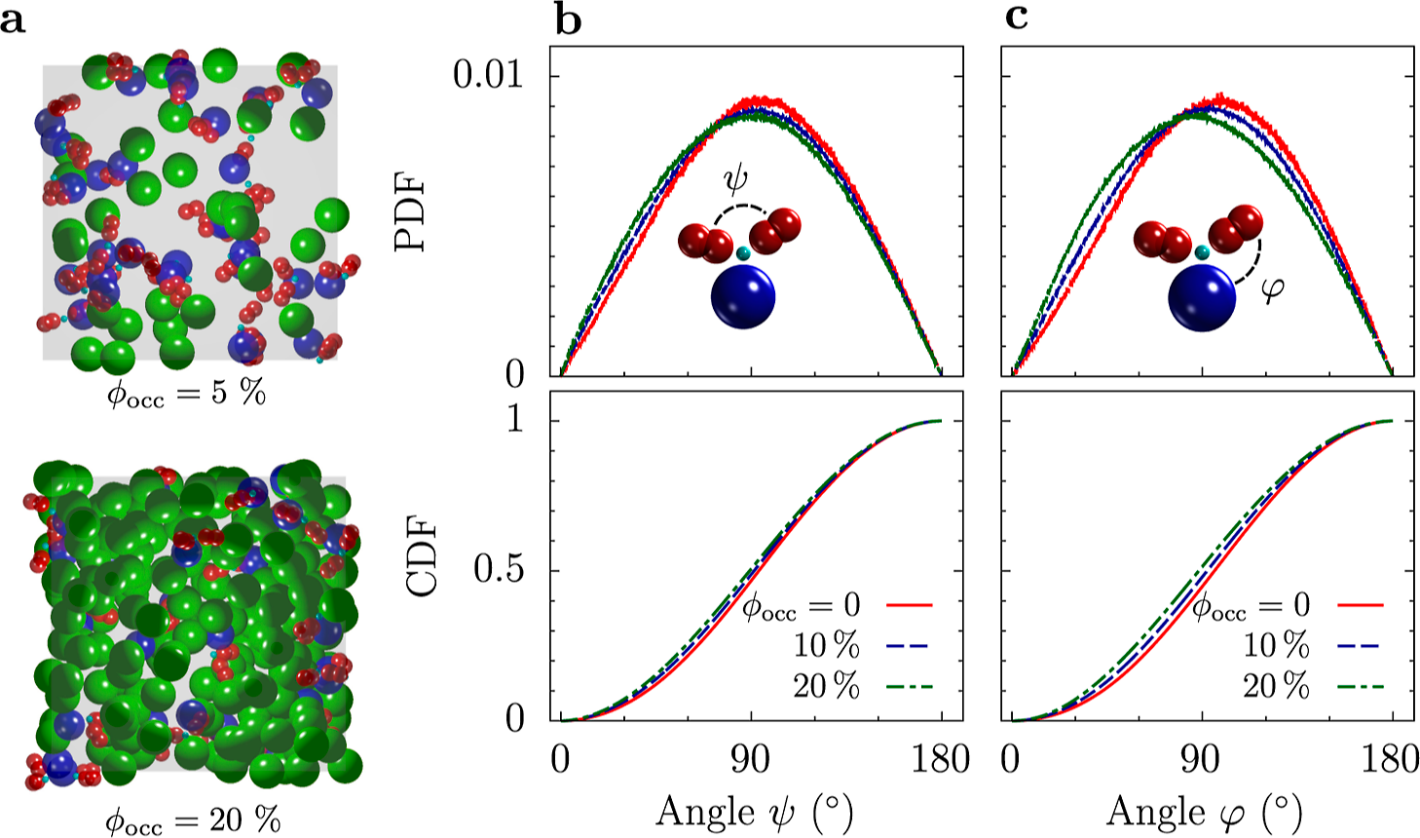
IgG’s flexibility under macromolecular crowding. (a) Snapshots from BD simulations of IgG–Ficoll mixtures for two values of the occupied volume fraction ϕ_occ_. (b) PDF (top) and CDF (bottom) of the angle ψ between the IgG arms for three values of ϕ_occ_, including a dilute system (ϕ_occ_ = 0). (c) PDF (top) and CDF (bottom) of the angle φ between the IgG stem and arms for three values of ϕ_occ_.

We first studied how crowding affects the distribution of angles. To this end, we performed BD simulations of mixtures of IgG and Ficoll 70 (Figure 3a), which is a typical synthetic crowder. Ficoll 70 was modeled as an uncharged spherical particle of radius 5.1 nm (we assumed the same hard-core and hydrodynamic radius). We fixed the number of IgG molecules in all simulations to gather sufficient statistics and increased crowding by adding Ficoll 70 (Section S1).

Figure 3b shows the PDF of the angle ψ between the arms and the corresponding cumulative distributions for two crowded systems. With increasing crowding, the distribution extends slightly to the left, and the peak shifts toward smaller angles because the crowders squeeze the IgG arms closer together. The angle φ between the arm and the stem also shifts toward smaller values, and the effect is even stronger than for ψ angles (Figure 3c). Overall, however, the effect of crowding is moderate, and the arms remain flexible even at an occupied volume fraction as high as 20%. It is worth noting that Girelli et al.^33^ also observed that an IgG preserves its high flexibility in solutions crowded with poly(ethylene glycol) (PEG) (6% weight/volume concentration). However, we note that PEGs are soft and can form polymeric networks at high concentrations,^34^ providing different crowding environments. Słyk et al.^35^ have demonstrated that diffusion in soft and hard crowders can differ substantially, particularly for non-spherical particles. It will be interesting to study in future work how IgG’s flexibility and diffusion depend on the type of crowders.

### Diffusion

**Figure 4:**
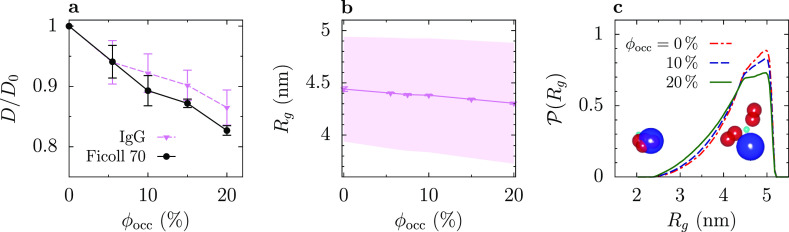
Diffusion of IgG. (a) Translational diffusion coefficient *D* expressed in terms of the diffusion coefficient in infinite dilution (*D*_0_) as a function of the occupied volume fraction ϕ_occ_ for IgG and Ficoll 70. (b) Gyration radius *R*_g_ of IgG as a function of ϕ_occ_. The shadowed area shows the standard deviation of the distribution of *R*_g_ [see (c)]. (c) Distribution of *R*_g_ for three values of ϕ_occ_. The snapshots show two typical configurations corresponding to large and small *R*_g_.

We next investigated the translational diffusion of IgG. In Figure 4a, we plot the long-time diffusion coefficients of Ficoll 70 and IgG as functions of the total occupied volume fraction ϕ_occ_. Although IgG’s hydrodynamic radius (*a*_H_ ≈ 5.8 nm) is larger than that of Ficoll 70 (5.1 nm), its diffusion coefficient is reduced less strongly with increasing ϕ_occ_. This is unexpected because crowders typically slow down the diffusion of larger macromolecules more strongly.^36,37^

To understand this result, we calculated the radius of gyration *R*_g_ of IgG in all crowded systems. Figure 4b presents *R*_g_ as a function of ϕ_occ_, and in Figure 4c, we plot the distribution of gyration radii for three values of ϕ_occ_, including infinite dilution (ϕ_occ_ = 0). These plots show that *R*_g_ decreases with ϕ_occ_ due to the suppression of most extended conformations (the increase in the number of compact structures is moderate and spans a broader range of gyration radii). Thus, the dependence of the diffusion coefficient on ϕ_occ_ is an interplay of two competing effects. On the one hand, the IgG diffusion slows down because crowders act as obstacles. On the other hand, they reduce the IgG size, thus enhancing its diffusion. To demonstrate this interplay, we assume *D* ≈ *k*_B_*T* (1 – αϕ_occ_)/(6πη*a*_H_(ϕ_occ_)), where η is the dynamic viscosity. Note that α depends on the size of tracer particles.^36,38^ Ignoring this dependence in the lowest order in ϕ_occ_ and taking a constant α = α_IgG_^nonflex^ (diffusion decrease rate assuming a non-flexible IgG of average size at zero crowding), one finds *D* ≈ *D*_0_ (1 – [α_IgG_^nonflex^–α_g_]ϕ_occ_), where we have used *a*_H_ ≈ *a*_H_(ϕ_occ_ = 0)(1 – α_g_ϕ_occ_), assuming that the hydrodynamic radius of IgG follows the same dependence on ϕ_occ_ as *R*_g_; fitting this equation to the simulation data in Figure 4b, we get α_g_ ≈ 0.17. Fitting now *D*(ϕ_occ_) = 1 – αϕ_occ_ to the simulation data in Figure 4a, we obtain α_Ficoll_ ≈ 0.89 for Ficoll and α_IgG_ ≈ 0.71 for IgG, the latter giving α_IgG_^nonflex^ = α_IgG_ + α_g_ ≈ 0.88. Although α_IgG_^nonflex^ is only comparable to α_Ficoll_ (we recall that IgG is slightly larger than Ficoll 70), this estimate demonstrates how the crowding-induced reduction in the IgG size can enhance its diffusion.

Since the rotational diffusion coefficient *D*_r_ increases as an inverse cube and *D* only inversely proportionally to the particle size, the reduced IgG size should affect *D*_r_ even more strongly than *D*. Yet, rotational diffusion per se is less affected by crowding than translational diffusion,^32,39,40^ making resolving the ϕ_occ_ dependence computationally more challenging. We extracted *D*_r_ through fitting to orientation autocorrelation functions (Section S1.4.2) and found that indeed *D*_r_ varies rather weakly with ϕ_occ_. However, the value of *D*_r_ was sensitive to statistics and the time window wherein the fitting was applied (Figure S1), preventing us from drawing definite conclusions about the dependence of *D*_r_ on ϕ_occ_. We leave such studies to future work.

### Binding Kinetics

#### First Binding Step

We now turn to the binding kinetics and investigate how the rate of the first binding step depends on crowding. To this end, we employed the Northrup–Allison–McCammon (NAM) approach,^41^ implemented in the pyBrown simulation package.^29^ Within this approach, the association rate between two molecules is computed from Brownian (or molecular) dynamics simulations of two reacting molecules by starting a simulation at separation *l*_0_ between the molecules and counting the number of reacting trajectories. A reaction counts as occurring when a certain reaction criterion (e.g., the distance between the molecules) is satisfied and not occurring when the particle separation becomes larger than a certain escape distance *l*_esc_. The reaction rate is then given by^41^
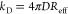
3where *D* is the mutual diffusion coefficient and
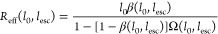
4is the effective reaction radius. Here, β is the percentage of reactive trajectories for given *l*_0_ and *l*_esc_ and Ω is the probability that the molecules at separation *l*_esc_ get back to separation *l*_0_, which for spherical, non-interacting particles is equal to *l*_0_/*l*_esc_. We note that both *D* and *R*_eff_ depend on crowding.

An IgG binds to an antigen by a paratope, a small antigen binding site located at the tip of each IgG arm. However, using such a binding criterion led to poor simulation statistics, requiring many independent simulation runs. Since we are solely interested in the effect of crowding on binding, for simplicity of calculations and to speed up our simulations, we used a criterion in which the *A*_2_ or *A*_2_′ unit was sufficiently close to an antigen (we chose the surface-to-surface distance of 0.1 nm). Consequently, we present only the ratio of the reaction constant to its value at zero crowding. For other parameters, we chose *l*_0_ = 30 nm and *l*_esc_ = 35 nm (see Figure S3 for a smaller value of *l*_esc_). For each occupied volume fraction ϕ_occ_, we performed at least 1000 independent simulation runs.

**Figure 5:**
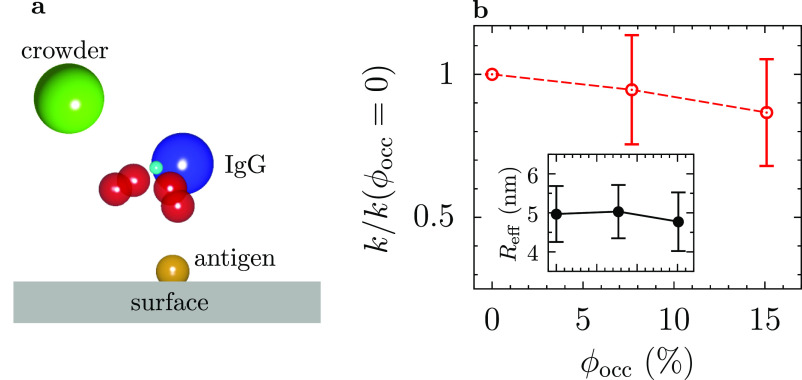
First step of the IgG–antigen binding. (a) Schematic of an IgG binding to an antigen on a surface. (b) Effect of crowding on reaction rate *k* of the IgG–antigen binding expressed in terms of the ratio of *k*(ϕ_occ_) to *k*(ϕ_occ_ = 0). The reaction rates were computed using the NAM algorithm.^41^ The inset shows the effective reaction radius of the NAM algorithm (eq 4), indicating that the reaction slowdown is driven by the IgG’s diffusion slowdown (see Figure 4a).

Figure 5b shows the reaction rate constant *k*(ϕ_occ_) divided by *k*(ϕ_occ_ = 0) and the inset shows the effective reaction radius *R*_eff_ computed according to eq 4. These plots indicate that the reaction proceeds slower under crowding and that the diffusion slowdown drives it. This behavior is likely because the IgG arms preserve their high flexibility under crowding (Figure 3), only slightly affecting the binding to an antigen once the IgG is at the surface.

#### Second Binding Step

**Figure 6:**
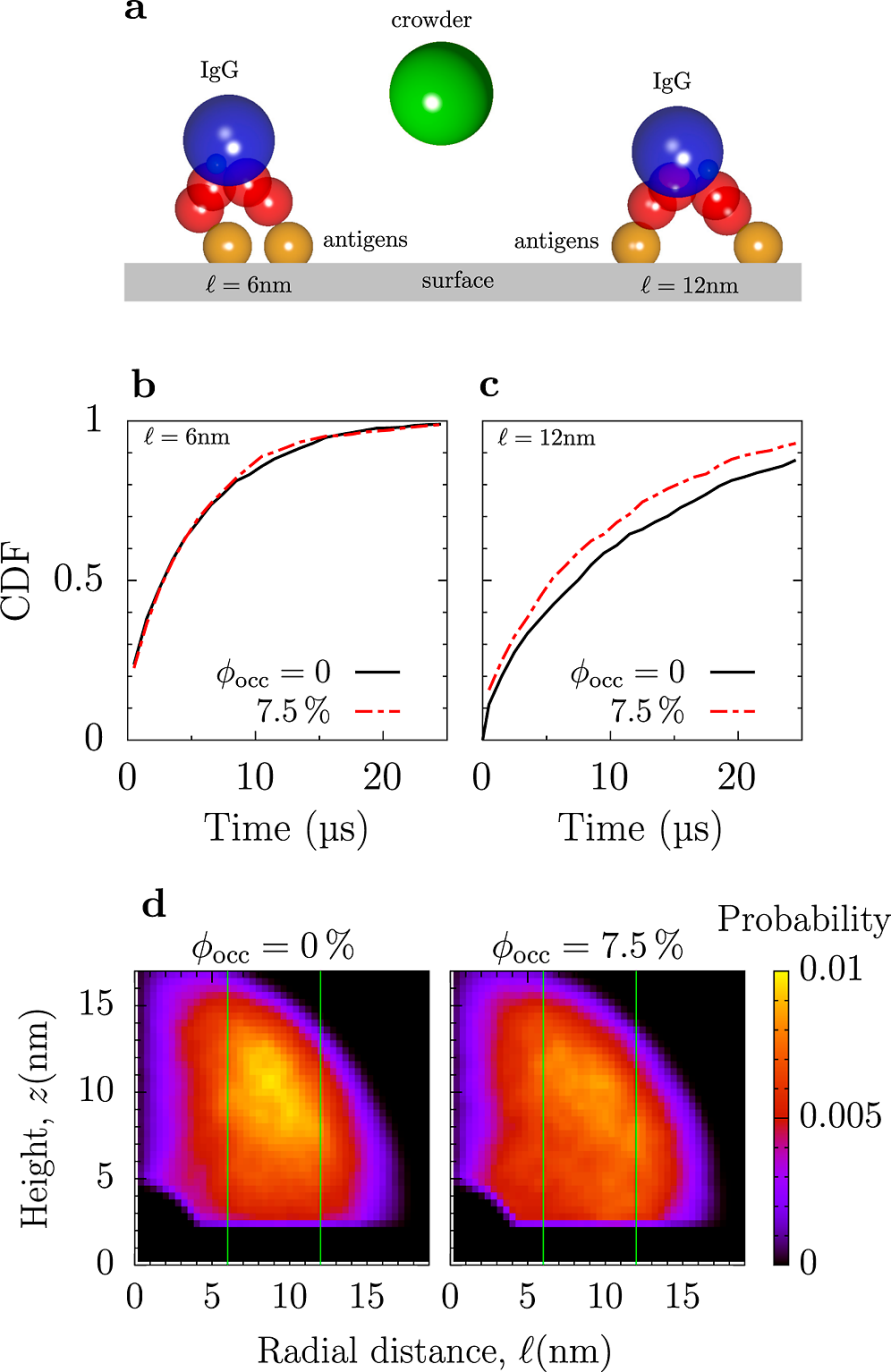
Second step of the IgG–antigen binding. (a) Schematic of an IgG bound to two antigens on a surface for antigen separation 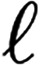. (b,c) Effect of crowding on cumulative distribution of times of IgG binding to the second antigen for two antigen separations. (d) Normalized distribution of heights over the surface and radial distances of the second IgG arm from the first (bound) antigen. The green vertical lines show the separations between the antigens used in (b,c).

To investigate how crowding affects the second binding step, we considered two antigens on a surface separated by a distance 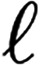 from each other and an IgG bound to one antigen with one of its arms (Figure 6a). We performed BD simulations for this setup, assuming the same binding criterion as for the first binding step, and computed the distribution of times when the IgG binds to the second antigen. Cumulative distributions are shown in Figure 6b,c for two values of 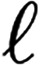. This figure shows that the binding occurs faster when the antigens are closer to each other, which is because for a smaller 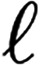, the second IgG arm has to explore a smaller area to find an antigen.

Unlike the first binding step, crowding enhances the second step, and the enhancement is stronger for larger antigen separations. To understand this result, we plot the distribution of heights *z* over the surface and radial distances 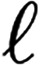 of the IgG arm from the first (bound) antigen (Figure 6d). At zero crowding, there is a peak plateau in the distribution around *z* ≈ 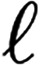 ≈ 10 nm (the yellowish region in Figure 6d). This peak disappears in the presence of crowders, and the distribution becomes more homogeneous. This behavior is likely because the crowders above the IgG promote the spreading of the IgG arms. Thus, crowding increases the probability of the IgG arm being closer to the surface at a larger 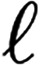, enhancing the second binding step. In this case, the influence of crowders is more substantial than in bulk, where we saw only minor changes in the IgG’s arm flexibility (Figure 3). This is because an IgG is bound by one of its arms to the surface, which enhances excluded volume effects.

## Conclusions

We have introduced a computationally inexpensive coarse-grained model of IgG, consisting of only six beads connected so as to mimic the IgG shape (Figure 1). We tuned the model parameters to reproduce the distribution of angles between the IgG arms and stem measured with cryo-electron tomography^13^ (Figure 2) and the translational diffusion of IgG measured in infinite dilution.^31^ The model can be applied to study processes where the detailed molecular structure of IgG is not essential or in hybrid simulations,^42,43^ combining it with less coarse models, e.g., of Galanti et al.^17^ We previously used this model to study diffusion in soft crowders^35^ and the thermodynamics of divalent binding.^44^ Further work may extend the model to include dispersion interactions and microscopic details of the binding process, for instance by adding explicitly binding sites (paratopes).

We used our IgG model in BD simulations to investigate how crowding affects the diffusion and binding of IgG to antigens in biologically relevant macromolecularly crowded media. We found that the IgG diffusion slows down less strongly with increasing crowding than the diffusion of Ficoll, even though the IgG is larger and expected to move slower under the same crowding conditions.^36,37^ We related this behavior to the IgG’s flexibility and crowding-induced conformational changes, which effectively reduce the IgG size as the concentration of crowders increases (Figure 4).

We found that crowding only weakly affects the distribution of angles between the IgG’s arms and stem (Figure 3). As a consequence, the binding of IgG to antigens on a surface is also slightly affected by crowding. We found that the reaction rate of the first binding step was reduced by about 12% for an occupied volume fraction of 15%. However, even this relatively weak slowdown was mainly due to the crowding-induced slowdown of IgG diffusion (Figure 5). In contrast to the first binding step, the second step was enhanced by crowding but also relatively weakly (Figure 6).

We hope this model and the results motivate further simulation and experimental studies on the behavior of IgG under physiologically relevant crowding conditions. The knowledge acquired with such studies will help us better understand our immune response system and may be useful to develop novel biomedical applications.

## Supporting Information Available

Details of Brownian dynamics simulations and the NAM algorithm for calculating reaction constants (PDF)
