# Supplementary material for: Minimal Coarse-Grained Model for Immunoglobulin G: Diffusion and Binding under Crowding
Source: J Phys Chem B. 2023 Aug 17;127(34):7442–8. doi: 10.1021/acs.jpcb.3c02383 (PMC10476189; doi:10.1021/acs.jpcb.3c02383)
Supplement: Supplementary file 1 — jp3c02383_si_001.pdf [file jp3c02383_si_001.pdf]

Supplementary Material to: “A Minimal Coarse-Grained Model for Immunoglobulin G (IgG): Diffusion and Binding under Crowding”

Edyta Słyk,[†,‡,‖] Tomasz Skóra,[†,¶,‖] and Svyatoslav Kondrat[*,†,§]

†*Institute of Physical Chemistry, Polish Academy of Sciences, 01-224 Warsaw, Poland*

‡*Department of Theoretical Chemistry, Institute of Chemical Sciences, Faculty of Chemistry, Maria Curie-Skłodowska University in Lublin, 20-031 Lublin, Poland*

¶*Current address: Scientific Computing and Imaging Institute, University of Utah, Salt Lake City, Utah 84112, United States*

§*Institute for Computational Physics, University of Stuttgart, Stuttgart 70569, Germany*

‖*Contributed equally to this work.*

E-mail: svyatoslav.kondrat@gmail.com;skondrat@ichf.edu.pl

Contents

S1 Brownian dynamics simulations **S3**

S1.1 Hydrodynamic interacions S4

S1.2 Repulsive interactions S4

S1.3 Simulation details S5

S1.4 Analysis S6

S1.4.1 Translational diffusion S6

S1.4.2 Rotational diffusion S7

S2 Reactions **S8**

S2.1 First binding step S8

S2.2 Second binding step S10

References **S12**

S1 Brownian dynamics simulations

We performed Brownian dynamics (BD)[1,2] simulations using the propagation scheme due to Iniesta and García de la Torre[3]:

$$\boldsymbol{r}(t+\Delta t) = \boldsymbol{r}(t) + \frac{1}{2}\frac{\Delta t}{k_{\mathrm{B}}T}\left[\boldsymbol{D}(t)\boldsymbol{F}(t) + \boldsymbol{D}\left(t+\frac{1}{2}\Delta t\right)\boldsymbol{F}\left(t+\frac{1}{2}\Delta t\right)\right] + \sqrt{\Delta t\left[\boldsymbol{D}\left(t\right) + \boldsymbol{D}\left(t+\frac{1}{2}\Delta t\right)\right]}\boldsymbol{X}, \quad \text{(S1)}$$

where t is time, Δt is propagation step, $\boldsymbol{r}$ is $3N$-dimensional vector of positions, N is number of beads, k_{B} is Boltzmann constant, T is temperature, $\boldsymbol{D}$ is $3N \times 3N$ diffusion matrix, $\boldsymbol{F}$ is $3N$-dimensional vector of forces, and $\boldsymbol{X}$ is $3N$-dimensional vector of mutually-independent random normally-distributed variables with zero mean and unit variance. Square root of a matrix is defined by its Choleski factor $\boldsymbol{B}$, *i.e.*,

$$\boldsymbol{D} = \boldsymbol{B}^T\boldsymbol{B}. \quad \text{(S2)}$$

To find the values of $\boldsymbol{D}$ and $\boldsymbol{F}$ at time $t + \Delta t/2$, simpler propagation scheme due to Ermak and McCammon[1] is used:

$$\boldsymbol{r}(t+\frac{\Delta t}{2}) = \boldsymbol{r}(t) + \frac{1}{2}\frac{\Delta t}{k_{\mathrm{B}}T}\boldsymbol{D}(t)\boldsymbol{F}(t) + \sqrt{\Delta t\boldsymbol{D}\left(t\right)}\boldsymbol{X}. \quad \text{(S3)}$$

As the $\boldsymbol{D}$ matrix changes slowly with time, for the sake of computational efficiency we recomputed $\boldsymbol{D}$ and $\boldsymbol{B}$ every 100 steps instead of every step, following other papers[4–7].

S1.1 Hydrodynamic interacions

We computed the position-dependent diffusion matrix $\boldsymbol{D}$ in a generalized Rotne-Prager-Yamakawa (RPY) approximation[8–11]:

$$\boldsymbol{D}_{ij}(r_{ij}) = \frac{k_\mathrm{B}T}{8\pi\eta r_{ij}}\left[\left(1+\frac{a_i^2+a_j^2}{3r_{ij}^2}\right)\boldsymbol{I}+\left(1-\frac{a_i^2+a_j^2}{r_{ij}^2}\right)\hat{\boldsymbol{r}}_{ij}\hat{\boldsymbol{r}}_{ij}^T\right] \tag{S4a}$$

for $r_{ij} > a_i + a_j$ and

$$\boldsymbol{D}_{ij}(r_{ij}) = \frac{k_\mathrm{B}T}{8\pi\eta r_{ij}}\left[\frac{16r_{ij}^3(a_i+a_j)-[(a_i-a_j)^2+3r_{ij}^2]^2}{32r_{ij}^3}\boldsymbol{I}+\frac{3[(a_i-a_j)^2-r_{ij}^2]^2}{32r_{ij}^3}\hat{\boldsymbol{r}}_{ij}\hat{\boldsymbol{r}}_{ij}^T\right] \tag{S4b}$$

for $a_{ij}^M - a_{ij}^m < r_{ij} < a_i + a_j$, where $a_{ij}^M = \max(a_i, a_j)$ and $a_{ij}^m = \min(a_i, a_j)$. Finally:

$$\boldsymbol{D}_{ij} = \frac{k_\mathrm{B}T}{6\pi\eta a_{ij}^M}\boldsymbol{I}, \tag{S4c}$$

for $r_{ij} < a_{ij}^M - a_{ij}^m$. Here η is fluid viscosity, $\hat{\boldsymbol{r}}_{ij}$ is normalized vector connecting i-th and j-th bead, r_{ij} is distance between them, and a_i is i-th bead's hydrodynamic radius.

To account for slow decay of $\boldsymbol{D}$ with distance ($\propto r_{ij}^{-1}$), we used Ewald summation scheme proposed by Smith *et al.*[12]. We set the parameter α controling the convergence of the Ewald summation to $\sqrt{\pi}$ (default value in `BD_BOX`). The maximal magnitude of both real (m_cutoff) and reciprocal (n_cutoff) lattice vectors was 2.

S1.2 Repulsive interactions

For two large spheres composed of small particles of radii equal to σ interacting with the repulsive component of the Lennard-Jones potential (LJ12) with energy ε_LJ, the total interaction potential is an integral of LJ12 potential over the volumes of the two spheres. Its polynomial expansion in r_{ij} was derived by Henderson *et al.*[13] and for small separations can

be approximated as follows[4]:

$$V_{\mathrm{rep}}\left(r_{ij}\right)=\frac{64\varepsilon_{\mathrm{LJ}}\pi^2}{315}\left(\frac{a_i a_j}{a_i+a_j}\right)\frac{\sigma^6}{\left[r_{ij}-\left(a_i+a_j\right)\right]^7}. \tag{S5}$$

Parameters for repulsive interactions are: $\varepsilon_{\mathrm{LJ}} = 0.37\,\mathrm{kcal\,mol^{-1}}$, $\sigma = 0.15\,\mathrm{nm}$. We included repulsive interactions between any pairs of particles, apart from the IgG beads belonging to the same macromolecule.

To avoid large forces causing numerical problems, for separations between the macromolecules' surfaces below $r_{\mathrm{min}} = 0.3\,\mathrm{nm}$, we kept the magnitude of force fixed and equal to $\boldsymbol{F}(r_{\mathrm{min}})$. The upper cutoff for the repulsive interactions was set to 25 nm. Following Ando and Skolnick[4], we accounted for macromolecules' roughness by multiplying potential from Eq. (S5) by:

$$\frac{r_{ij}-\left(a_i+a_j\right)}{r_{ij}-\left(a_i+a_j\right)+0.5\left(h_i+h_j\right)}, \tag{S6}$$

setting $h = 0.94\,\mathrm{nm}$ for Ficoll70, 1.21 nm for S, 0.30 nm for H, 0.73 nm for A_1, A_1', A_2, and A_2'.

S1.3 Simulation details

We performed BD simulations with a customized version of the `BD_BOX` software[14,15]. The customization concerned allowing for overlaps between bonded beads and performing diffusion matrix computation and Choleski decoposition every 100 steps, instead of every step, to decrease the computation time.

BD was performed in cubic boxes of $85\,\mathrm{nm} \times 85\,\mathrm{nm} \times 85\,\mathrm{nm}$, and we applied periodic boundary conditions in all three directions. Time step Δt was 0.5 ps and simulations lasted for at least 15 µs. Temperature was 298.15 K and dynamic viscosity was $\eta = 1.02\,\mathrm{cP}$.

The compositions of mixtures studied in this work (*i.e.*, molar fractions and number of particles) are shown in Table S1.

Table S1: Composition of studied mixtures

Volume fraction	Molar fraction		Molecule number	
	IgG	Ficoll	IgG	Ficoll
5.65 %	0.50	0.50	30	30
7.64 %	0.37	0.63	30	52
10.08 %	0.28	0.72	30	79
15.06 %	0.18	0.82	30	134
19.95 %	0.14	0.86	30	188

S1.4 Analysis

In every analysis procedure we discarded first 1 µs of the trajectory to ensure that the system is equilibrated.

S1.4.1 Translational diffusion

We obtained translational diffusion coefficients from Time-Averaged Mean Squared Displacement (TAMSD):

$$\mathrm{TAMSD}(m\Delta t) = \frac{1}{N_{\mathrm{traj}}}\sum_{i=1}^{N_{\mathrm{traj}}}\frac{1}{N_{\mathrm{steps}}-m}\sum_{k=0}^{N_{\mathrm{steps}}-m-1}\{\boldsymbol{r}_i\left[(k+m)\Delta t\right]-\boldsymbol{r}_i\left[k\Delta t\right]\}^T\{\boldsymbol{r}_i\left[(k+m)\Delta t\right]-\boldsymbol{r}_i\left[k\Delta t\right]\}, \tag{S7}$$

where $\Delta t = 10\,\mathrm{ns}$ is a window length, $\boldsymbol{r}_i$ is position vector of i-th bead, N_{traj} is the number of trajectories, and N_{steps} is the number of steps in a trajectory. The long-time diffusion coefficients were obtained by averaging D/D_0 between 3 and 5 µs. Uncertainty of the D_l due to sampling error was estimated by dividing the simulations into 5 subsets and computing standard deviation of the mean, while treating the subsets as independent "measurements".

S1.4.2 Rotational diffusion

We obtained rotational diffusion coefficients from Orientation Autocorrelation (OA)[16,17]:

$$\mathrm{OA}(m\Delta t) = \frac{1}{N_{\mathrm{traj}}} \sum_{i=1}^{N_{\mathrm{traj}}} \frac{1}{N_{\mathrm{steps}} - m} \sum_{k=0}^{N_{\mathrm{steps}}-m-1} \boldsymbol{\ell}_i\left((k+m)\,\Delta t\right) \cdot \boldsymbol{\ell}_i\left(k\Delta t\right), \tag{S8}$$

where $\boldsymbol{\ell}$ is the orientation vector and the window length Δt is the same as in Eq. (S7). We defined orientation by a vector connecting S with A_2, but we also verified that alternatives: S-H and A_1-A_2, lead to similar results. OA decays exponentially with time, and the decay rate is related to the rotational diffusion coefficient D_r[16]:

$$\mathrm{OA}(t) = \exp\left(-2D_r t\right). \tag{S9}$$

We obtained D_r by fitting exponential decay to the obtained OA between 0 and 2 µs. The resulting D_r's are shown in Fig. S1.

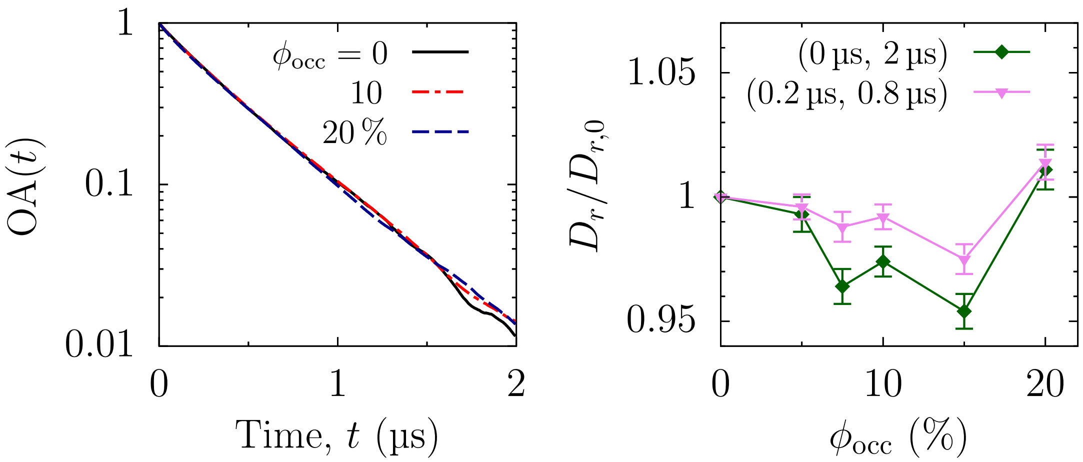

Figure S1: **Macromolecular crowding and rotational diffusion of IgG.** (left) Orientation autocorrelation function *vs.* time for three values of the occupied volume fraction ϕ_{occ} measured for the SA_2 segment of IgG. (right) Rotational diffusion coefficient D_r as a function of ϕ_{occ} extracted from OA using Eq. (S9). The curves are calculated for two time intervals used for fitting, as indicated on the plot. D_r is expressed in terms of the rotational diffusion coefficient in infinite dilution ($D_{r,0}$). Rotational diffusion coefficient depends only weakly on ϕ_{occ}, but its value is sensitive to the time interval where the fitting is applied.

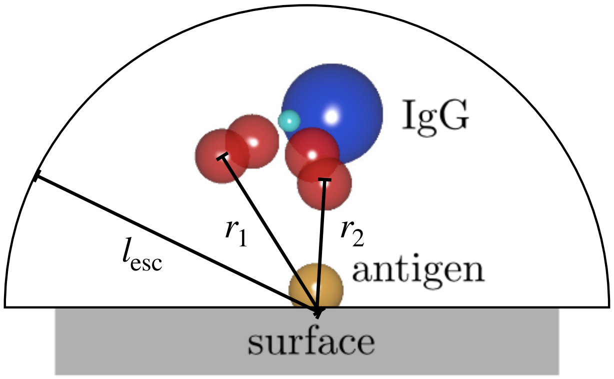

Figure S2: **Simulations of antibody-antigen diffusion-controlled binding kinetics.** Distance between i-th arm and antigen is denoted by r_i. If any r_i becomes smaller than r_{react}, the trajectory is considered *reactive*. If distance l between hinge and antigen gets larger than l_{esc}, the trajectory is considered *nonreactive*.

S2 Reactions

S2.1 First binding step

Diffusion-controlled reaction rates were computed using modified Northrup-Allison-McCammon (NAM) algorithm[18] implemented in `pyBrown` Brownian dynamics package[19] (`https://tskora.github.io/pyBrown/`). It is based on the BD simulations in which system consists of a single IgG macromolecule and a single antigen *hot spot* of radius equal to 2.42 nm, however the difference from the original algorithm is that the center of the antigen is placed on an impenetrable surface (see Fig. S2). As the approximations for including hydrodynamic interactions in BD simulations don't account for interactions with walls or immobile particles, we neglect hydrodynamic interactions in our BD-NAM simulations.

The simulations are started from configurations in which the IgG center of geometry is at a distance l_0 from antigen along the plane normal vector. The initial orientation and internal configuration of IgG are drawn from Boltzmann distribution by sampling from separate BD

Table S2: **Compositions of BD systems used to investigate the first IgG-antigen binding step.**

ϕ_{occ} (%)	N_{IgG}	N_{F}	l_0 (nm)	l_{esc} (nm)	V (nm^3)
0		0	20	25	
7.6	1	21	20	25	$75 \times 75 \times 37.5$
15.2		42	20	25	
0		0	30	35	
7.7	1	29	30	35	$75 \times 75 \times 47.5$
15.1		57	30	35	

simulations of a system composed of a single IgG molecule. Single BD-NAM simulation is terminated when one of two events happens:

1. one of the arms of IgG gets very close to the antigen hot spot ($r_i < 4.94\,\mathrm{nm}$, $i \in \{1,2\}$) ,
2. the hinge gets very far from the antigen hot spot ($l > l_{\mathrm{esc}} = 25$ or $35\,\mathrm{nm}$).

Then we use the expression obtained by Northrup, Allison, and McCammon[18] to express the bimolecular diffusion-controlled reaction rate constant k_D using the percentage of reactive trajectories β for a given l_0 and l_{esc}. Assuming that all collisions are reactive, the expression reads[18]:

$$k_D = 4\pi D l_0 \frac{\beta}{1-(1-\beta)\,\Omega} = 4\pi D R_{\mathrm{eff}}, \tag{S10}$$

where Ω is probability that molecule with $l = l_{\mathrm{esc}}$ gets back to l_0. Assuming that particles at long distances are well approximated by noninteracting (neither hydrodynamically nor directly) spheres, $\Omega = l_0/l_{\mathrm{esc}}$.

We performed BD-NAM simulations for $\phi_{\mathrm{occ}} = 0$, ≈ 7.5, and $\approx 15\%$. Compositions of the systems are gathered in Table S2. We applied periodic boundary conditions in x and y directions, but in the z direction, we enclosed the system between two impenetrable walls. Thus, when computing ϕ_{occ}, we divide the occupied volume by the volume *accessible* to

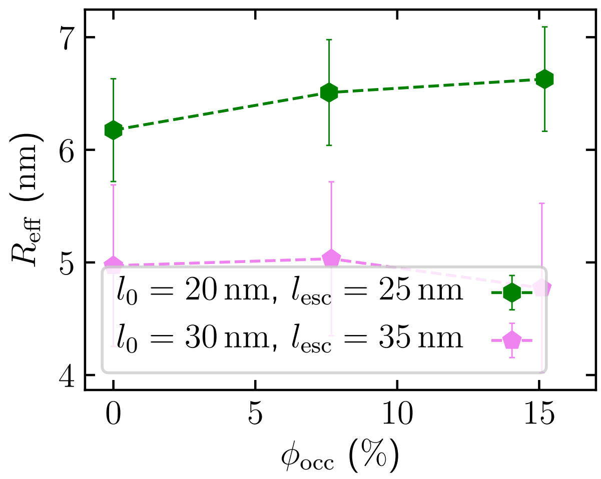

Figure S3: **Antibody-antigen diffusion-controlled kinetic rate *vs.* crowding.**

them, *i.e.*:

$$\phi_{\mathrm{occ}} = \frac{4}{3}\pi a_{\mathrm{cr}}^3 \frac{N_{\mathrm{cr}}}{L_x \times L_y \times (L_z - 2a_{\mathrm{cr}})}. \tag{S11}$$

For every system, we ran 800-1000 simulations. Uncertainties due to sampling errors were estimated by dividing the simulations into five subsets and computing the standard deviation of the mean. The NAM results obtained using Eq. (S10) are presented in Fig. S3.

The details of BD simulations are as follows. The time step Δt was 0.5 ps. BD was propagated with the Ermak and McCammon scheme (Eq. (S3), but with $\Delta t/2 \to \Delta t$). The temperature T was set to 293.15 K, and dynamic viscosity to $\eta = 1.005$ cP. The crowder particles interacted with each other and with IgG only with a hard-sphere potential.

S2.2 Second binding step

Subsequently, we used BD simulations to inspect the kinetics of the second step of IgG-antigen binding. We placed additional antigen on the surface in distance of 6, 9, and 12 nm from the first one. Compositions of the systems are gathered in Table S3. We measured how much time it takes IgG bound with one arm to one antigen to bind to the second antigen with

Table S3: **Compositions of BD systems used to investigate the second IgG-antigen binding step.**

ϕ_{occ} (%)	N_{IgG}	N_{F}	l_{agene} (nm)	V (nm^3)
0	1	0	6	$75 \times 75 \times 37.5$
0		0	9	
0		0	12	
7.5		21	6	
7.5		21	9	
7.5		21	12	

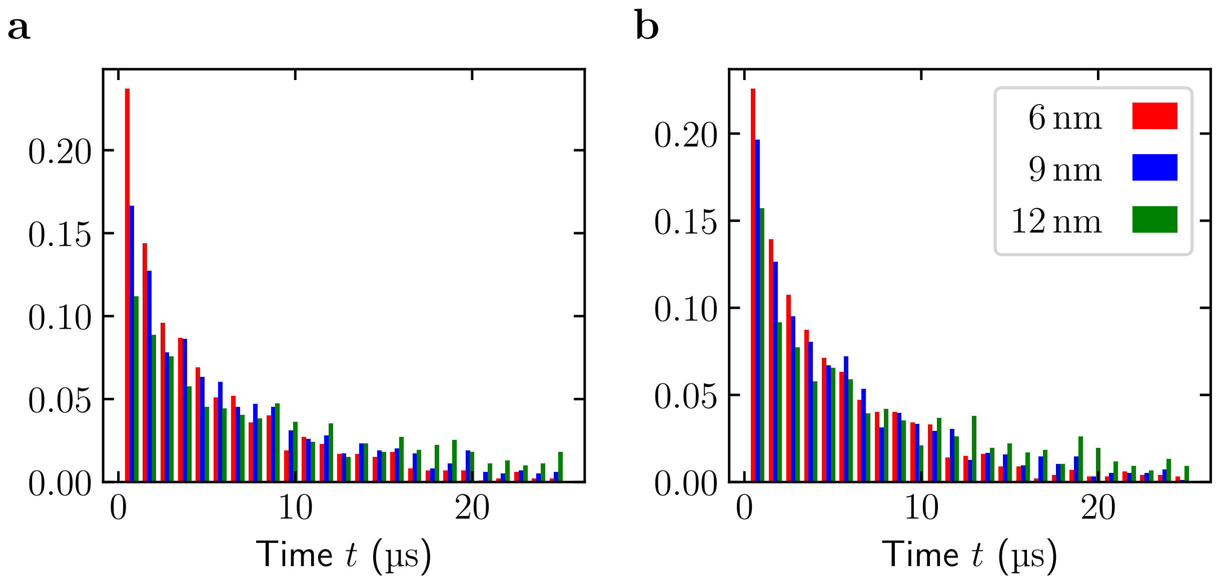

Figure S4: **Second step of antibody-antigen binding: reaction times in function of antigen-antigen distance.** (**a**) $\phi_{\mathrm{occ}} = 0\%$. (**b**) $\phi_{\mathrm{occ}} = 7.5\%$.

its free arm. Starting conformations were sampled from separate Brownian dynamics simulation of IgG bound with 1 arm to a single antigen. Bonded potential between arm tip and antigen was the same as between A_1 and A_2, *i.e.*, $r_{\mathrm{eq}} = 3.0$ nm and 1909.9 kcal mol^{-1} nm^{-2}. For each system, 750-1000 simulations were performed. Histograms of reaction times are presented in Fig. S4, and the cumulative distribution functions in Fig. S5.

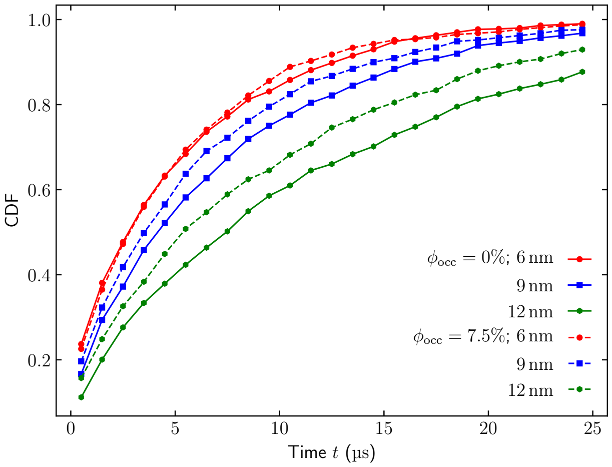

Figure S5: **Second step of antibody-antigen binding: cumulative distribution functions.**

References

(1) Ermak, D. L.; McCammon, J. A. Brownian dynamics with hydrodynamic interactions. *J. Chem. Phys.* **1978**, *69*, 1352–1360.

(2) Huber, G. A.; McCammon, J. A. Brownian Dynamics Simulations of Biological Molecules. *Trends Chem.* **2019**, *1*, 727–738.

(3) Iniesta, A.; García de la Torre, J. A second-order algorithm for the simulation of the Brownian dynamics of macromolecular models. *J. Chem. Phys.* **1990**, *92*, 2015–2018.

(4) Ando, T.; Skolnick, J. Crowding and hydrodynamic interactions likely dominate in vivo macromolecular motion. *Proc. Natl. Acad. Sci.* **2010**, *107*, 18457–18462.

(5) Skóra, T.; Vaghefikia, F.; Fitter, J.; Kondrat, S. Macromolecular Crowding: How Shape and Interactions Affect Diffusion. *J. Phys. Chem. B* **2020**, *124*, 7537–7543.

(6) Skóra, T.; Popescu, M. N.; Kondrat, S. Conformation-changing enzymes and macromolecular crowding. *Phys. Chem. Chem. Phys.* **2021**, *23*, 9065–9069.

(7) Słyk, E.; Skóra, T.; Kondrat, S. How macromolecules softness affects diffusion under crowding. *Soft Matter* **2022**, *18*, 5366–5370.

(8) Rotne, J.; Prager, S. Variational Treatment of Hydrodynamic Interaction in Polymers. *J. Chem. Phys.* **1969**, *50*, 4831–4837.

(9) Yamakawa, H. Transport properties of polymer chains in dilute solution: hydrodynamic interaction. *J. Chem. Phys.* **1970**, *53*, 436–443.

(10) De La Torre, J. G.; Bloomfield, V. A. Hydrodynamic properties of macromolecular complexes. I. Translation. *Biopolymers* **1977**, *16*, 1747–1763.

(11) Zuk, P. J.; Wajnryb, E.; Mizerski, K. A.; Szymczak, P. Rotne-Prager-Yamakawa approximation for different-sized particles in application to macromolecular bead models. *J. Fluid Mech.* **2014**, *741*, R5.

(12) Smith, E. R.; Snook, I. K.; Van Megen, W. Hydrodynamic interactions in Brownian dynamics. *Phys. A Stat. Mech. its Appl.* **1987**, *143*, 441–467.

(13) Henderson, D.; Duh, D.-M.; Chu, X.; Wasan, D. An expression for the dispersion force between colloidal particles. *J. Colloid Interface Sci.* **1997**, *185*, 265–268.

(14) BDBOX. `https://www.fuw.edu.pl/{~}mdlugosz/downloads.html`.

(15) Długosz, M.; Zieliński, P.; Trylska, J. Brownian dynamics simulations on CPU and GPU with BD-BOX. *J. Comput. Chem.* **2011**, *32*, 2734–2744.

(16) Kharazmi, A.; Priezjev, N. V. Molecular Dynamics Simulations of the Rotational and Translational Diffusion of a Janus Rod-Shaped Nanoparticle. *J. Phys. Chem. B* **2017**, *121*, 7133–7139.

(17) Balbo, J.; Mereghetti, P.; Herten, D.-P.; Wade, R. C. The Shape of Protein Crowders is a Major Determinant of Protein Diffusion. *Biophys. J.* **2013**, *104*, 1576–1584.

(18) Northrup, S. H.; Allison, S. A.; McCammon, J. A. Brownian dynamics simulation of diffusion-influenced bimolecular reactions. *J. Chem. Phys.* **1984**, *80*, 1517–1524.

(19) Skóra, T.; Kondrat, S. pyBrown: Versatile Brownian and Stokesian Dynamics Package for Simulations of Diffusion and Reactions. *in preparation* **2023**,
